# Supplementary material for: Corticostriatal control of defense behavior in mice induced by auditory looming cues
Source: Nat Commun. 2021 Feb 15;12:1040. doi: 10.1038/s41467-021-21248-7 (PMC7884702; doi:10.1038/s41467-021-21248-7)
Supplement: Supplementary file 1 — Supplementary Information [file 41467_2021_21248_MOESM1_ESM.pdf]

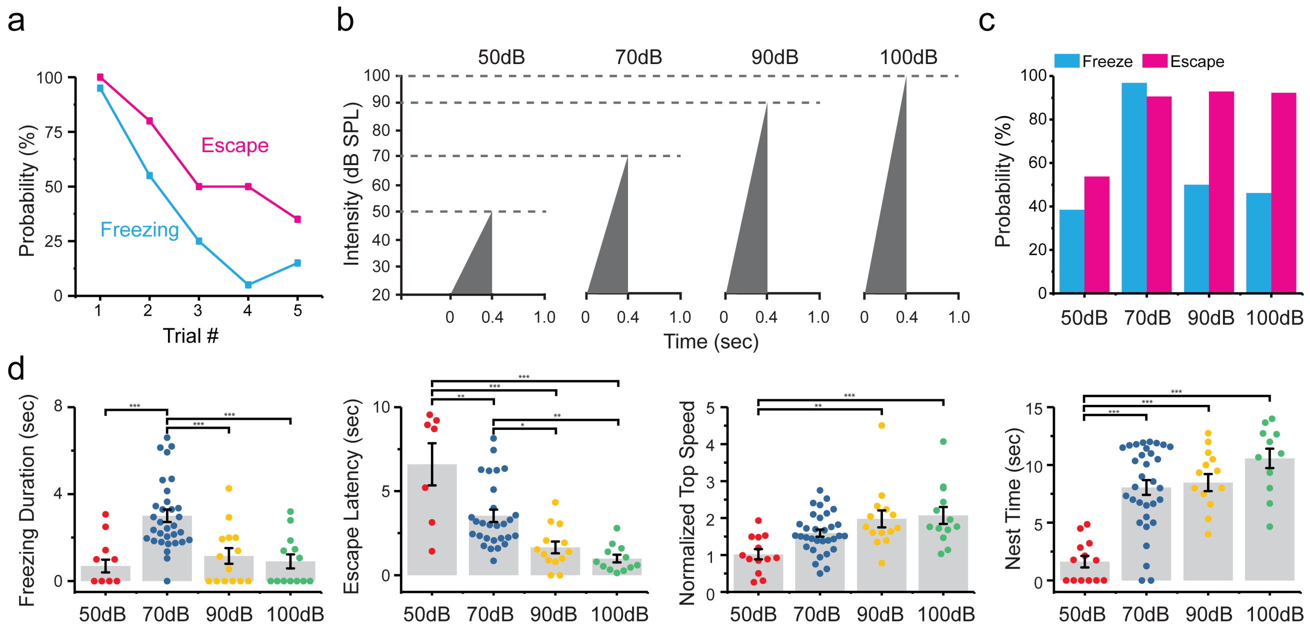

Supplementary Fig. 1. Impact of sound intensity on induced behaviors. **a**, Probability of animal exhibiting freezing (blue) or escape (red) behavior over 5 consecutive trials. **b**, Illustration of crescendo stimuli with different top intensities. **c**, Probability of freezing (blue) and escape (red) induced by crescendo stimuli of different top intensities (from left to right: n = 13, 32, 14 and 13 mice, respectively). **d**, Comparison of freezing duration, escape latency, normalized top speed and nest time between crescendo of different top intensities. Freezing duration, n = 13, 32, 14, 13; 50dB vs. 70dB, ***p = 2.606 x 10-5; 70dB vs. 90dB, ***p = 7.202 x 10-4; 70dB vs. 100dB, ***p = 1.498 x 10-4. Escape latency, n = 7, 28, 13, 12; 50dB vs. 70dB, **p = 0.002; 50dB vs. 90dB, ***p = 3.250 x 10-6; 50dB vs. 100dB, ***p = 2.675 x 10-7; 70dB vs. 90dB, *p = 0.024; 70dB vs. 100dB, **p = 0.001. Normalized top speed, n = 13, 32, 14, 13; 50dB vs. 90dB, **p = 0.002; 50dB vs. 100dB, ***p = 8.273 x 10-4. Nest time, n = 13, 32, 14, 13; 50dB vs. 70dB, ***p = 1.413 x 10-7; 50dB vs. 90dB, ***p = 1.409 x 10-6; 50dB vs. 100dB, ***p = 1.808 x 10-9. One-way ANOVA with post hoc (Bonferroni) test.

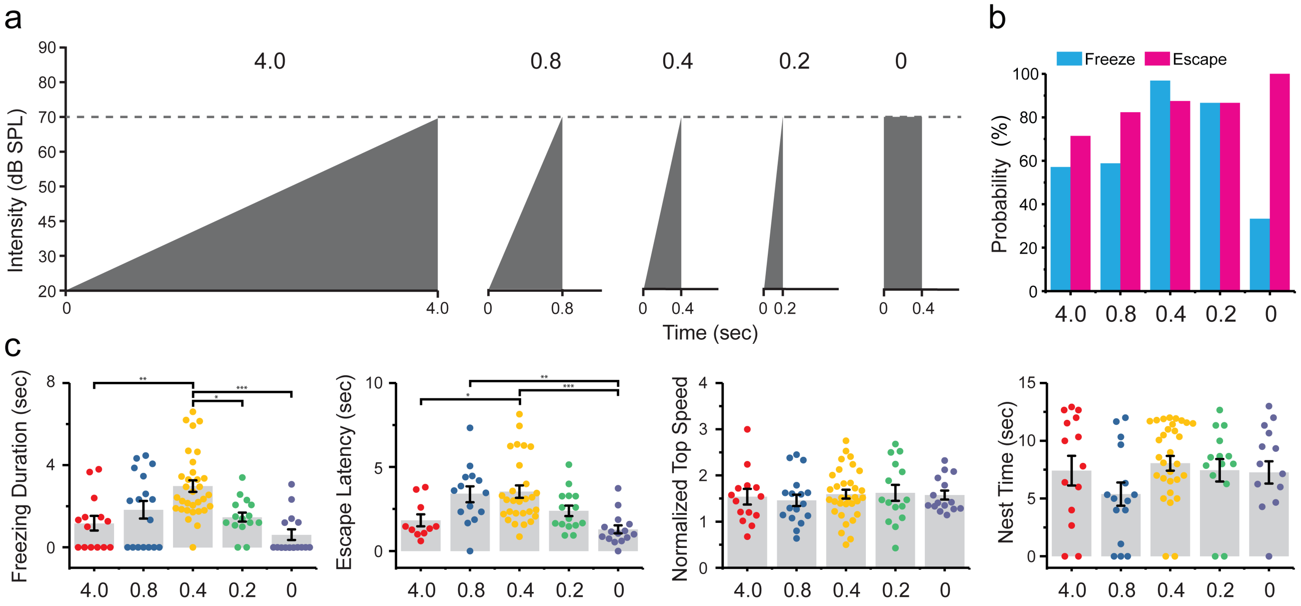

Supplementary Fig. 2. Impact of rise time on induced behaviors. a, Illustration of crescendo stimuli with different rise times. **b**, Probability of freezing (blue) and escape (red) induced by crescendo stimuli of different rise times (from left to right: n = 14, 17, 32, 15 and 15 mice, respectively). **c**, Comparison of freezing duration, escape latency, normalized top speed and nest time between crescendo of different rise times. Freezing duration, n = 14, 17, 32, 15, 15; 4.0s vs. 0.4s, **p = 0.001; 0.4s vs. 0.2s, *p = 0.013; 0.4s vs 0s, ***P = 7.713 x 10-6. Escape latency, n = 14, 14, 28, 13, 15; 4.0s vs 0.4s, *p = 0.042; 0.8s vs. 0s, **p = 0.006; 0.4s vs. 0s, ***p = 2.701 x 10-4. Normalized top speed, n = 14, 17, 32, 15, 15. Nest time, n = 14, 17, 32, 15, 15. One-way ANOVA with post hoc (Bonferroni) test.

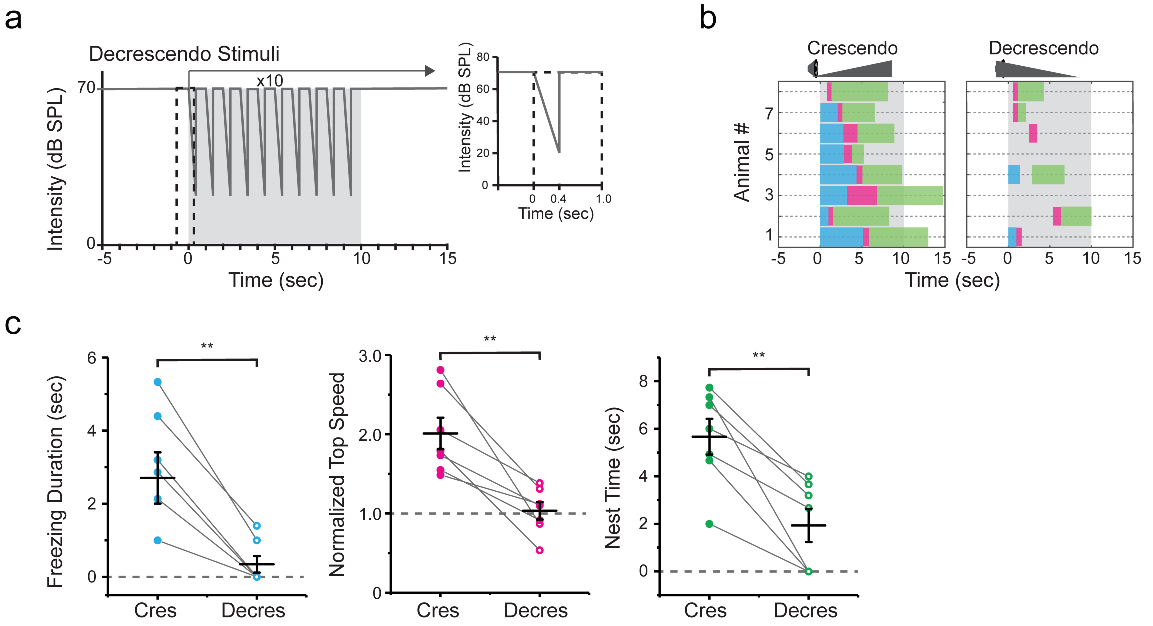

Supplementary Fig. 3. Decrescendo stimuli with high-level background fail to induce defense behaviors. **a**, Illustration of decrescendo stimuli with 70 dB background. Right, one individual stimulus. **b**, Comparison of behavioral responses to crescendo and decrescendo stimuli in the same animals (n = 8 mice). **c**, Comparison of freezing duration (left, **p = 0.002, two-sided paired t test, t = 4.667), normalized top speed (middle, **p = 0.001, two-sided paired t test, t = 4.580) and nest time (right, **p = 0.006, two-sided paired t test, t = 3.315) induced by crescendo (Cres.) and decrescendo (Decres.) stimuli in the same animals (n = 8 mice). Data are presented as mean ± s.e.m. The center presents the mean value and the bar represents s.e.m.

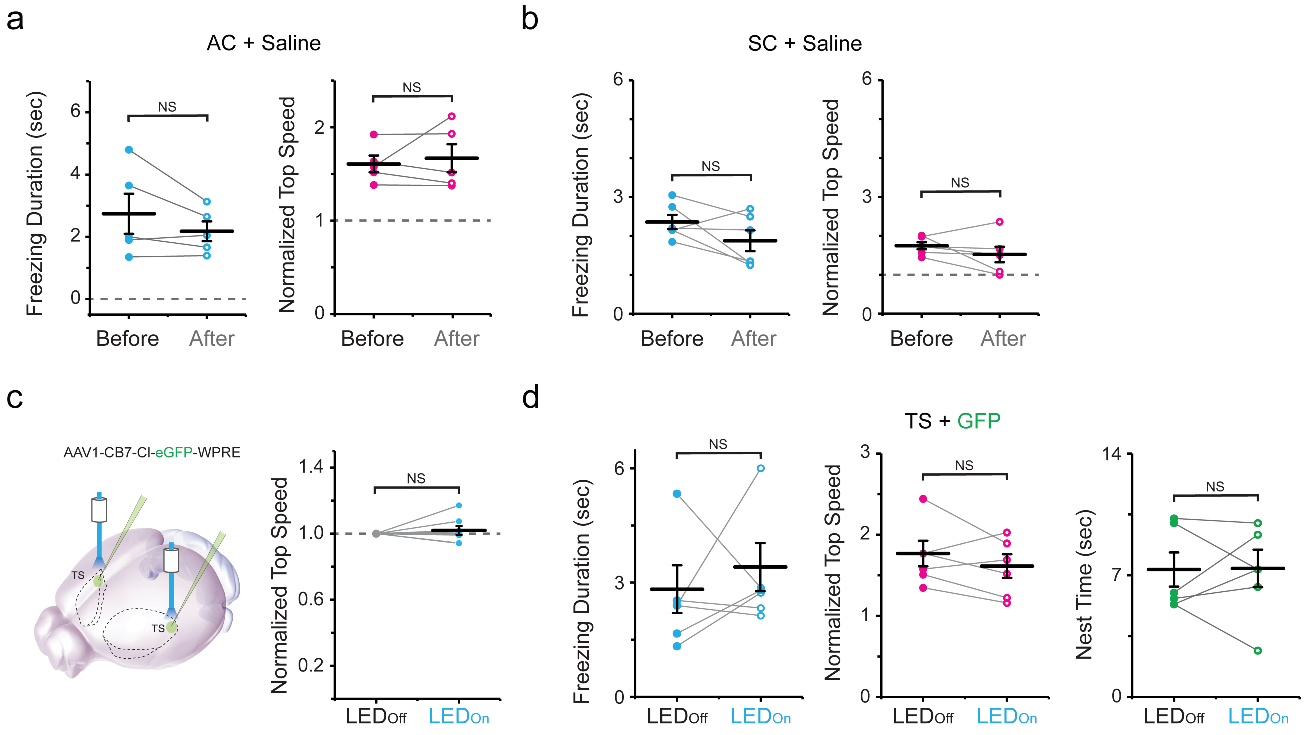

Supplementary Fig. 4. Control groups for AC, SC and TS manipulations. **a**, Quantitation of crescendo-induced freezing duration (left, p = 0.09, two-sided paired t test, t = 1.635) and normalized top speed (right, p = 0.68, two-sided paired t test, t = -0.496) before and after injection of saline in the auditory cortex (AC, n = 5 mice). **b**, Crescendo-induced freezing duration (left, p = 0.07, two-sided paired t test, t = 1.720) and normalized top speed (right, p = 0.13, two-sided paired t test, t = 1.264) before and after injection of saline in the superior colliculus (SC, n = 6 mice). **c**, No effect of LED stimulation on locomotion in mice with AAV-GFP injected in TS (p = 0.72, two-sided paired t test, t = -0.624, n = 8 mice). **d**, No effects of LED stimulation on crescendo-induced freezing duration (left, p = 0.73, two-sided paired t test, t = -0.642), normalized top speed (right, p = 0.13, two-sided paired t test, t = 1.257) or nest time (right, p = 0.52, two-sided paired t test, t = -0.069) in mice with AAV-GFP injected in TS (n = 6 mice). Data are presented as mean ± s.e.m. for **a-d**. The center presents the mean value and the bar represents s.e.m.

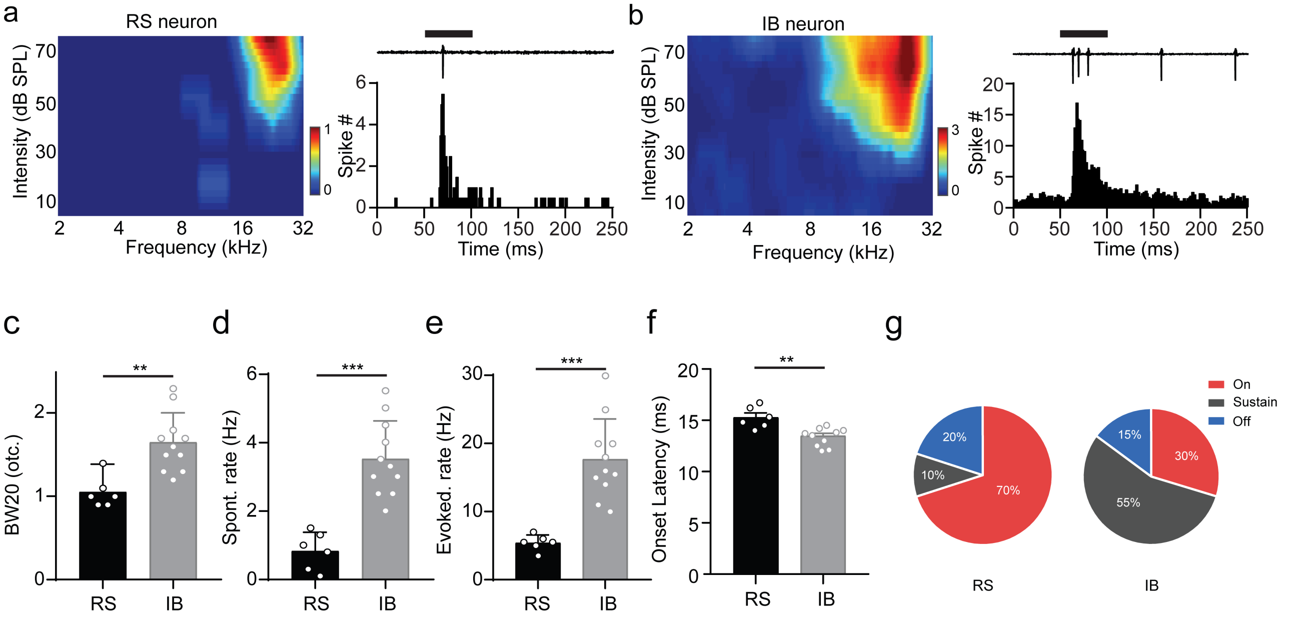

Supplementary Fig. 5. Response properties of regular spiking (RS) and intrinsic bursting (IB) neurons in layer 5 of auditory cortex (AC). a, Left, frequency-intensity tonal receptive field (TRF) of an example RS neuron. Color scale represents the number of spikes evoked. Right, peri-stimulus spike time histogram (PSTH) of the neuron's responses to best-frequency tones. Top inset, example recorded trace. Note that only one spike was evoked. **b**, An example IB neuron. Note that a train of spikes was evoked. **c-f**, Comparison of bandwidth of TRF at 20 dB above the intensity threshold (i.e. BW20, **c**), spontaneous firing rate (**d**), evoked firing rate (**e**) and onset latency (**f**) between RS (n = 6) and IB (n = 11) neurons. BW20: **p = 0.0017, t = -3.81; Spontaneous firing rate: ***p = 5.718 x 10-5, t = -5.535; Evoked firing rate: ***p = 1.680 x 10-4, t = -4.969; Onset latency: **p = 0.002, t = -3.750, two-sided t test. **g**, Proportions of ON, OFF and sustained types in the RS and IB groups. Data are presented as mean + s.e.m in **c-f**. The vertical bar represents s.e.m.
