# Supplementary material for: Corticostriatal control of defense behavior in mice induced by auditory looming cues
Source: Nat Commun. 2021 Feb 15;12:1040. doi: 10.1038/s41467-021-21248-7 (PMC7884702; doi:10.1038/s41467-021-21248-7)
Supplement: Supplementary file 3 — Description of Additional Supplementary Files [file 41467_2021_21248_MOESM3_ESM.docx]

**Description of Additional Supplementary Files**

File Name: Supplementary Movie 1

Description: Crescendo-induced defense behavior. 5-s crescendo was applied to a mouse in open-field test.

File Name: Supplementary Movie 2

Description: Effect of auditory cortices (AC) sliencing on defense behaviors. Left, control group; Right, muscimol group.

File Name: Supplementary Movie 3

Description: Effect of superior colliculus (SC) sliencing on defense behaviors. Left, control group; Right, muscimol group.

File Name: Supplementary Movie 4

Description: Optogenetic activation of the tail of the striatum (TS). Left, ChR2 group; Right, GFP group.

File Name: Supplementary Movie 5

Description: Optogenetic inactivation of the tail of the striatum (TS). Left, HR3.0 group; Right, GFP group.
