# Supplementary material for: Corticostriatal control of defense behavior in mice induced by auditory looming cues
Source: Nat Commun. 2021 Feb 15;12:1040. doi: 10.1038/s41467-021-21248-7 (PMC7884702; doi:10.1038/s41467-021-21248-7)
Supplement: Supplementary file 5 — Reporting Summary [file 41467_2021_21248_MOESM5_ESM.pdf]

nature research

Corresponding author(s): Li I. Zhang

Last updated by author(s): Dec 14, 2020

Reporting Summary

Nature Research wishes to improve the reproducibility of the work that we publish. This form provides structure for consistency and transparency in reporting. For further information on Nature Research policies, see our Editorial Policies and the Editorial Policy Checklist.

Statistics

For all statistical analyses, confirm that the following items are present in the figure legend, table legend, main text, or Methods section.

n/a	Confirmed	
☐	☒	The exact sample size (n) for each experimental group/condition, given as a discrete number and unit of measurement
☐	☒	A statement on whether measurements were taken from distinct samples or whether the same sample was measured repeatedly
☐	☒	The statistical test(s) used AND whether they are one- or two-sided<br>*Only common tests should be described solely by name; describe more complex techniques in the Methods section.*
☒	☐	A description of all covariates tested
☐	☒	A description of any assumptions or corrections, such as tests of normality and adjustment for multiple comparisons
☐	☒	A full description of the statistical parameters including central tendency (e.g. means) or other basic estimates (e.g. regression coefficient) AND variation (e.g. standard deviation) or associated estimates of uncertainty (e.g. confidence intervals)
☐	☒	For null hypothesis testing, the test statistic (e.g. F, t, r) with confidence intervals, effect sizes, degrees of freedom and P value noted<br>*Give P values as exact values whenever suitable.*
☒	☐	For Bayesian analysis, information on the choice of priors and Markov chain Monte Carlo settings
☒	☐	For hierarchical and complex designs, identification of the appropriate level for tests and full reporting of outcomes
☐	☒	Estimates of effect sizes (e.g. Cohen's d, Pearson's r), indicating how they were calculated

Our web collection on statistics for biologists contains articles on many of the points above.

Software and code

Policy information about availability of computer code

Data collection	Behavioral videos were recorded by OBS studio 25.0 (OBS project); Electrophysiological data was acquired by Open Ephys system 0.4.6 (Open Ephys); LabVIEW 2012.
Data analysis	Behavioral tracking data was analyzed by Tracker 5.1.0 (OSP); Sorting of electrophysiological data was performed by Offline Sorter 4.4.0 (Plexon) and analyzed with Matlab R2018a (Mathworks); Statistics was performed using Origin Pro 2017 (OriginLab); Histological images were analyzed using ImageJ 1.52i (Fiji).

For manuscripts utilizing custom algorithms or software that are central to the research but not yet described in published literature, software must be made available to editors and reviewers. We strongly encourage code deposition in a community repository (e.g. GitHub). See the Nature Research guidelines for submitting code & software for further information.

Data

Policy information about availability of data

All manuscripts must include a data availability statement. This statement should provide the following information, where applicable:

- Accession codes, unique identifiers, or web links for publicly available datasets
- A list of figures that have associated raw data
- A description of any restrictions on data availability

Source data are provided with this paper. All the data of the figures (not including anatomical images) in this study are provided as a source data file. Anatomical images are available from the corresponding authors upon reasonable request. Analysis codes used in this study are available at GitHub (https://github.com/ZhongliUSC/Looming-Sound) and archived in Zenodo (Zhong, L. et al. Corticostriatal Control of Defense Behavior in Mice Induced by Auditory Looming Cues. Looming-Sound 1.0 doi:10.5281/zenodo.4319461, 2020). Allen Brain Atlas (http://www.brain-map.org).

Field-specific reporting

Please select the one below that is the best fit for your research. If you are not sure, read the appropriate sections before making your selection.

☒ Life sciences ☐ Behavioural & social sciences ☐ Ecological, evolutionary & environmental sciences

For a reference copy of the document with all sections, see nature.com/documents/nr-reporting-summary-flat.pdf

Life sciences study design

All studies must disclose on these points even when the disclosure is negative.

Sample size	In the behavioral experiments, sample sizes were selected based on previous related experiments and the literature and verified by power analysis. A power analysis was also used to determine sample sizes in the electrophysiological recordings. The sample sizes of anatomical and RNAscope results were the maximal available of our dataset.
Data exclusions	All related data are included in analysis. There is no exclusion of data in this study.
Replication	Each experiment was successfully repeated for at least two more times.
Randomization	Animals were randomly assigned to control and treatment groups. For the animals with multiple treatment, the sequence of treatment was randomized.
Blinding	The investigators were not blinded to group allocation during behavioral and electrophysiological data collection, since the investigators must adjust and trigger different auditory stimuli. Data analyses were performed blinding to experimental conditions. For anatomical experiments, the investigators were not blinded to group allocation during data collection and/or analysis as no grouping in this study.

Reporting for specific materials, systems and methods

We require information from authors about some types of materials, experimental systems and methods used in many studies. Here, indicate whether each material, system or method listed is relevant to your study. If you are not sure if a list item applies to your research, read the appropriate section before selecting a response.

Materials & experimental systems

n/a	Involved in the study	
☒	☐	Antibodies
☒	☐	Eukaryotic cell lines
☒	☐	Palaeontology and archaeology
☐	☒	Animals and other organisms
☒	☐	Human research participants
☒	☐	Clinical data
☒	☐	Dual use research of concern

Methods

n/a	Involved in the study	
☒	☐	ChIP-seq
☒	☐	Flow cytometry
☒	☐	MRI-based neuroimaging

Animals and other organisms

Policy information about studies involving animals; ARRIVE guidelines recommended for reporting animal research

Laboratory animals	C57BL/6J: obtained from Jackson Laboratory (RRID:IMSR_JAX:000664), male and female, 2-3 months age<br>Ai14: obtained from Jackson Laboratory (RRID:IMSR_JAX:007914), male and female, 2-3 months age<br>Mice were group-housed in 18-23˚C with 40-60% humidity and a 12 hr light-dark cycle (light on: 6:00 a.m. to 6:00 p.m.).
Wild animals	The study did not involve wild animals.
Field-collected samples	The study did not involve samples collected from the field.
Ethics oversight	All experimental procedures in this study have been approved by the Institutional Animal Care and Use Committee (IACUC) of the University of Southern California.

Note that full information on the approval of the study protocol must also be provided in the manuscript.
